# *MDM2 *gene SNP309 T/G and *p53 *gene SNP72 G/C do not influence diffuse large B-cell non-Hodgkin lymphoma onset or survival in central European Caucasians

**DOI:** 10.1186/1471-2407-8-116

**Published:** 2008-04-23

**Authors:** Joerg Bittenbring, Frédérique Parisot, Alain Wabo, Monika Mueller, Lynn Kerschenmeyer, Markus Kreuz, Lorenz Truemper, Olfert Landt, Alain Menzel, Michael Pfreundschuh, Klaus Roemer

**Affiliations:** 1 Internal Medicine I and Josè Carreras Research Center, University of Saarland Medical School, Homburg-Saar, Germany; 2 Laboratoires Réunis, Junglister, Luxembourg; 3 University of Leipzig, Institute for Medical Informatics, Statistics and Epidemiology, Leipzig, Germany; 4 Hematology and Oncology, Medical School of the Georg-August-University, Göttingen, Germany; 5 TIB MOLBIOL GmbH, Berlin, Germany

## Abstract

**Background:**

SNP309 T/G (rs2279744) causes higher levels of MDM2, the most important negative regulator of the p53 tumor suppressor. SNP72 G/C (rs1042522) gives rise to a p53 protein with a greatly reduced capacity to induce apoptosis. Both polymorphisms have been implicated in cancer. The SNP309 G-allele has recently been reported to accelerate diffuse large B-cell lymphoma (DLBCL) formation in pre-menopausal women and suggested to constitute a genetic basis for estrogen affecting human tumorigenesis. Here we asked whether SNP309 and SNP72 are associated with DLBCL in women and are correlated with age of onset, diagnosis, or patient's survival.

**Methods:**

SNP309 and SNP72 were PCR-genotyped in a case-control study that included 512 controls and 311 patients diagnosed with aggressive NHL. Of these, 205 were diagnosed with DLBCL.

**Results:**

The age of onset was similar in men and women. The control and patients group showed similar SNP309 and SNP72 genotype frequencies. Importantly and in contrast to the previous findings, similar genotype frequencies were observed in female patients diagnosed by 51 years of age and those diagnosed later. Specifically, 3/20 female DLBCL patients diagnosed by 51 years of age were homozygous for SNP309 G and 2/20 DLBCL females in that age group were homozygous for SNP72 C. Neither SNP309 nor SNP72 had a significant influence on event-free and overall survival in multivariate analyses.

**Conclusion:**

In contrast to the previous study on Ashkenazi Jewish Caucasians, DLBCL in pre-menopausal women of central European Caucasian ethnicity was not associated with SNP309 G. Neither SNP309 nor SNP72 seem to be correlated with age of onset, diagnosis, or survival of patients.

## Background

The p53 tumor suppressor can drive stressed cells into senescence or apoptosis. One of the key negative regulators that keeps p53 in check in unstressed cells and limits p53's response under stress is the E3 ubiquitin ligase MDM2 [1]. A disequilibrium in the levels of MDM2 and p53 is associated with distinct phenotypes. For example, reduction of MDM2 expression in mice reduces adenoma formation [2] whereas MDM2 deficiency causing overshooting p53 activity was reported to be lethal [3,4]. On the other hand, overproduction of MDM2 is accompanied by a reduction of p53 activity and is a hallmark of some tumor types in humans [5-7]. Thus, inherited differences in the efficacy of the MDM2-mediated limitation of p53-response in stressed cells could be important determinants of efficient tumor suppression [8].

Intracellular MDM2 expression is controlled at the levels of protein stability, gene transcription, and transcript translation [1]. Upon stress or hormonal signalling, various transcription factors, among them p53 and the estrogen receptor ER-α [9] bind to response elements of the *MDM2 *gene promoter in the first intron. As a result, MDM2 levels rise and p53 activity is limited. Work by Bond and colleagues [10-12] has recently indicated that a single nucleotide polymorphism at intron 1 position 309 (rs2279744) generates a novel binding site for the ubiquitous transcriptional activator SP1 and causes higher MDM2 levels and consequently, attenuated p53 response in stressed or estrogen-exposed cells.

The *p53 *allele with a "C" instead of "G" at position 12139 (SNP72 C; rs1042522), coding for proline instead of arginine at amino acid position 72, occurs at a frequency of approximately 23% among Caucasians and is considered to be associated with at least some types of cancers [13]. Observations by Hong and colleagues suggest that homozygosity for both SNP309 G and SNP72 C can be additive [14]. The present study analyzes both polymorphisms in 311 patients with B-NHL and 512 healthy central Europeans of Caucasian ethnicity.

## Methods

### Study population

The cohort consisted of 311 patients from whom genomic DNA-samples were available that had biopsy-confirmed, aggressive NHL according to the Revised European-American Lymphoma Classification (translated into the World Health Organisation classification) and were treated in the NHL-B1 and B2 study [15,16] of the German High Grade Non-Hodgkin's lymphoma study group (DSHNHL). A subgroup of these patients was diagnosed with diffuse-large B-cell lymphoma (DLBCL; n = 205). Patients were excluded from the study if the diagnosis of aggressive or very aggressive lymphoma was not confirmed or if the diagnosis was changed into indolent lymphoma or no lymphoma at all by a panel of five expert hematopathologists in a blinded central pathology review. Other criteria for exclusion are summarized elsewhere [15,16]. Table 1 outlines the clinico-pathological characteristics and table 2 the histopathological diagnoses of the patients. Blood donors (n = 512) from the Institute for Transfusion Medicine, University of Saarland Medical School, served as controls. DNA from patients diagnosed with B-NHL was collected at the University of Göttingen during the study period.

**Table 1:** Clinico-pathological characteristics of the patients

		analyzed	
Patients characteristics	all trial patients B 1/2 (n = 1399)	NHL patients (n = 311)	DLBCL patients (n = 205)
Age Median; yr (range) 75)	60 (18–75)	62 (23–75)	61 (23–75)
Sex			
male	789 (56%)	175 (56%)	115 (56%)
female	610 (44%)	136 (44%)	90 (44%)
International Prognostic Index (IPI)			
Low (0,1)	840 (60%)	176 (57%)	118 (58%)
Low intermediate (2)	250 (18%)	62 (20%)	42 (20%)
High intermediate (3)	170 (12%)	46 (15%)	31 (15%)
High (4,5)	139 (10%)	27 (9%)	14 (7%)
Risk Age			
Age ≤ 60 yrs	710 (51%)	143 (46%)	98 (48%)
Age > 60 yrs	689 (49%)	168 (54%)	107(52%)
Risk extranodal involvement			
≤ 1 ex. involvement	1123 (80%)	254 (82%)	174(85%)
> 1 ex. involvement	276 (20%)	57 (18%)	31(15%)
Risk ECOG			
ECOG 0,1	1236 (88%)	273 (88%)	178(87%)
ECOG 2–4	163 (12%)	38 (12%)	27(13%)
Risk Stage			
Stage I, II	832 (59%)	191 (61%)	134(65%)
Stage III-IV	567 (41%)	120 (39%)	71(35%)
Risk LDH			
LDH ≤ ONW	1083 (77%)	241 (77%)	160(78%)
LDH > ONW	316 (23%)	70 (23%)	45(22%)
Bulky tumor (7.5 cm or larger)	467 (33%)	90 (29%)	62(30%)

**Table 2:** Histopathological characteristics

**number**	**(%)**	**Histopathological diagnosis (REAL-classification)**
284	91.32	**B-cell lymphomas**
205	65.92	**Diffuse large B-cell lymphoma**
13	4,18	DLBCL, NOS
8	2.57	anaplastic large-cell (ALC)
46	14.79	centroblastic diffuse, NOS
16	5.14	centroblastic diffuse, NOS -> monomorphic
9	2.89	centroblastic diffuse, NOS -> multi-lobulated
89	28.62	centroblastic diffuse, NOS -> polymorphic
17	5.47	immunoblastic
4	1.29	primary mediastinal B-cell lymphoma
3	0.96	T-cell rich B-cell-lymphoma
79	25.40	**Non-DLBCL B-cell lymphomas**
15	4.82	centroblastic-follicular
6	1.93	centroblastic follicular and diffuse
4	1.29	mantle-cell blastic variant
3	0.96	Burkitt-lymphoma
12	3.86	high-grade Burkitt-like
5	1.61	blastic marginal-zone
16	5.14	not otherwise specified
18	5.79	unclassified (technical reasons)
27	8.68	**T-cell lymphomas**
20	6.43	anaplastic large-cell
4	1.29	peripheral NOS -> small and large-cell
1	0.32	T-cell-lymphoma (AILD)
2	0.64	not otherwise specified

### DNA extraction and genotyping

Genomic DNA was isolated from whole blood with the QIAamp Blood Kit (Qiagen, Hilden). DNA was diluted in water to a final concentration of 15 ng/μl to use 5 μl (45 ng) per reaction. The mutation tests were performed in the LightCycler 1.2 (p53) or LightCycler 480 (MDM2) instrument, using the FastStart DNA Master Hybridization Probes kit with 3 mM MgCl_2 _(Roche Diagnostics, Mannheim) in a total volume of 20 μl, and analyzing the melting curve of the hybridization probes releasing from the PCR product. The analysis for the p53 codon 72 mutation was performed as described [17]. For the detection of the Mdm2 polymorphism rs2279744, we used 0.5 μM of the the primers mdmF^mt ^5'ggCTgCggggC**T**gCT-3'(position 2565–2579 in Genbank AF527840), changing base 2575C to T (underlined), and primer mdmR 5'-CCAATCCCgCCCAgACTAC-3'(2611–2637), plus 0,25 μM of the detection probes, consisting of the 3'-terminal fluorescein-labeled Sensor(T) C**T**gCT**T**CggCgCg_gATgATCgCAg–FL (position 2575(---)2607), specific for the T allele (underlined) also containing the base 2575T and a gap for the target sequence positions 2588–2596, and the 5'-LightCycler Red 640 labeled and 3'-phosphorylated anchor probe 640-CCTgTCgggTCACTAgTgTgAACgCTg–PH (2611–2637)(TIB MOLBIOL, Berlin). After an initial denaturation at 95°C for 12 min 30 s, amplification was performed using 45 cycles of denaturation (95°C, 5 sec, ramp rate 4.4°C), annealing (60°C, 10 sec, ramp rate 2.2), and extension (72°C, 20 sec, ramp rate 4.4°C). Fluorescence was measured at the end of the annealing period of each cycle. After the amplification a melting curve was generated: the PCR mixture was heated to 95°C for 20 sec, ramp rate 4.4°C/s, cooled to 40°C, 20 sec, ramp rate 1.5°C/sec and then slowly heated to 85°C with one acquisition per °C. The fluorescence signal was monitored continuously during the temperature ramp and then plotted against the temperature. These curves were transformed to derived melting curves (-d(F2)/dT vs. T).

### Design of MDM2 detection probes

The MDM2 target sequence is extremely rich in strong GC bases (70%). This is known to cause difficulties in PCR amplification. In particular, we found stem loop CCGC–GCGG, spanning the polymorphism, to have a Tm of 82°C (OLIGO 6.0, MBI). This stem loop was excluded from the PCR fragment by introduction of one base substitution C2575T which had to be changed also in the sensor probe. The segment around the mutated base contains only G and C bases (85%), making a probe based analysis difficult. A very short probe will fail to bind the mismatched allele whereas a longer probe will reach extremely high binding temperatures, causing poor differentiation of the variants in the melting curve analysis. To overcome these difficulties we followed the procedures of [18], introducing a 9 nucleotides gap ggaggtccg in the sensor probe, resulting in a 24 mer sequence with a GC content of 67%.

### Statistical analysis

Out of the NHL B1/2 trial patients, 311 DNA samples were available for analysis. The allelic frequencies were compared with Fisher's exact test. Mann-Whitney U test was used to test for differences among the onset of NHL by age. Event-free survival (EFS) was defined as the time from the beginning of therapy to either disease progression; initiation of salvage therapy; or additional (off-protocol) treatment, relapse, or death. Overall survival (OS) was defined as the time from first day of treatment to death from any cause. In EFS and OS analyses, a Cox multivariate analysis was done to adjust for known adverse risk factors, defined by the International Prognostic Index IPI, and additionally, for bulky disease (tumors > 7.5 cm anywhere). EFS and OS were estimated with the Kaplan-Meier method and were compared with the log-rank test. Differences between groups were regarded as significant for p values less than 0.05 (two-sided). Statistical analyses were performed with R 2.5.1. [19].

## Results

### MDM2 gene SNP309 T/G

**Figure 1:**
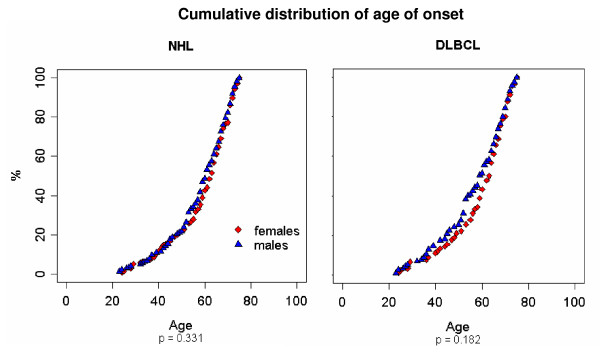
**NHL and DLBCL cumulative distribution of age of onset for men (triangles) and women (diamonds).** Age of onset was compared between male and female patients using the Mann-Whitney U test.

Among the 311 patients diagnosed with NHL, no difference in the onset by age between genders was detectable (p = 0.33). Specifically, male patients were diagnosed, on average, at the age of 58 years (range: 23–75 years) and female patients at the age of 59 years (range: 24–75 years)(Fig. 1). Similarly, in the subgroup of 205 patients diagnosed with DLBCL, no difference in the onset by age was detectable (p = 0.18). Men were diagnosed at the age of 57 years (range: 23–75 years) and women at the age of 59 years (range: 24–75 years)(figure 1).

**Figure 2:**
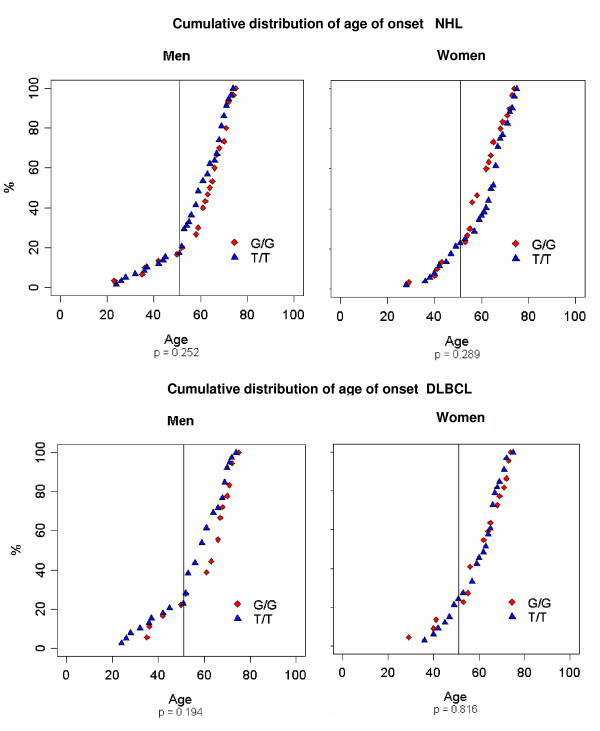
**NHL and DLBCL cumulative distribution of age of onset for males and females with the SNP309 G/G or T/T genotype.** Age of onset was compared between male and female patients using the Mann-Whitney U test.

**Figure 3:**
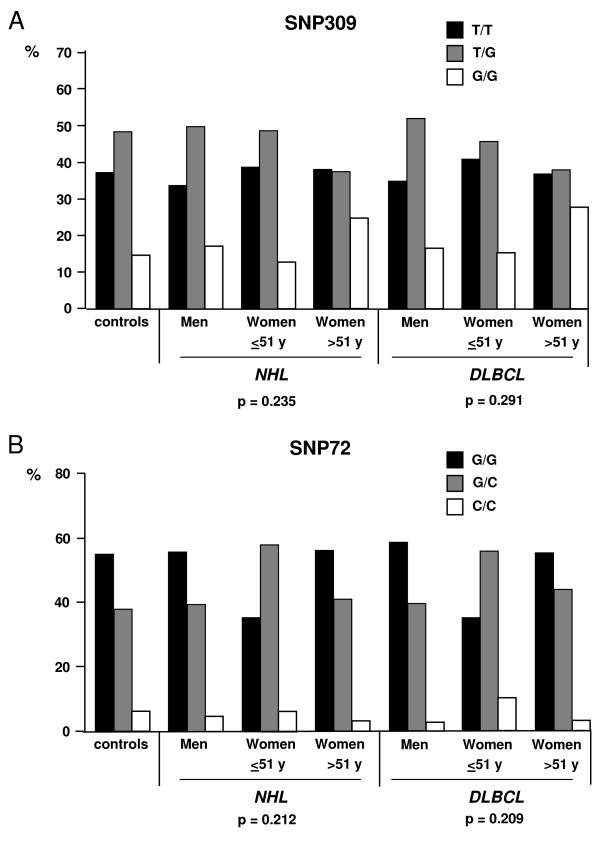
**Relative ratios of the SNP309 genotypes (A) and SNP72 genotypes (B) for the healthy controls, male NHL or DLBCL patients, and female NHL or DLBCL patients diagnosed by 51 years of age or later.** P-values were determined with Fisher's exact test (within patient samples).

Among 512 healthy central Europeans, 14% were homozygous for SNP309 G, and in the cohort of 311 central European patients diagnosed with NHL, 19% were homozygous; the genotype frequencies did not deviate from those expected under the Hardy-Weinberg equilibrium. Stratification according to SNP309 T/G genotypes failed to reveal differences in the onset of NHL by age in men (p = 0.25) and women (p = 0.29), and also in the onset of DLBCL by age in men (p = 0.19) and women (p = 0.82) (figure 2). Similar genotype frequencies were observed in the controls and the male NHL and DLBCL patient groups (figure 3). Previous work by Bond and colleagues had indicated that estrogen signalling can co-operate with the G-allele of SNP309 in lymphomagenesis in women, documented by stratification of the cohort in pre-menopausal women up to 51 years and older women [11]. In our study, no significant difference was detected between female NHL or DLBCL patients diagnosed by 51 years of age and those diagnosed later (figure 3A). Specifically, among all NHL patients, four of 31 women diagnosed by 51 years, and 26 of 105 women diagnosed later, exhibited the G/G genotype (p = 0.35). For women diagnosed with DLBCL, these numbers were 3/20 and 19/70, respectively (p = 0.59).

**Figure 4:**
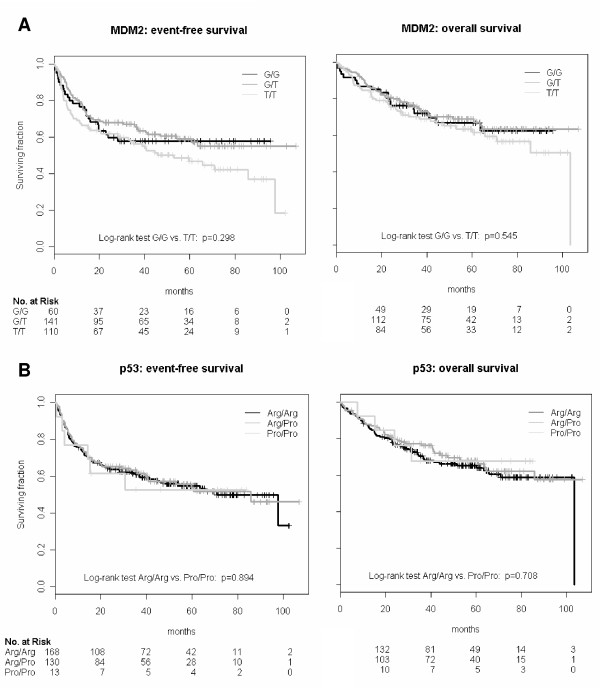
Kaplan-Meier plots for EFS and OS for the MDM2-SNP309 genotypes (A) and the p53 SNP 72 genotypes (B) in all NHL-patients.

Next, we examined whether SNP309 T/G can influence prognosis. For this purpose, Kaplan-Meier plots for EFS and OS, stratified according to genotypes, were calculated (figure 4A). Cox proportional hazard analysis to adjust for IPI-factors (age >60; ex. involvement >1; ECOG 2–4; Stage III-IV; LDH > ONV) and bulky disease showed no difference between the genotype groups G/G and T/T. (An independent influence of SNP309 T/G on EFS/OS could not be detected in this multivariate analysis). The results of the Cox regression analyses are summarized in Table 3.

**Table 3:** Cox regression adjusted for IPI-factors and bulky disease

*MDM2 *gene SNP309				
	event-free survival		overall survival	

	G/G vs. G/T and T/T	T/T vs. G/T and G/G	G/G vs. G/T and T/T	T/T vs. G/T and G/G

DLBC samples	1.20 (0.71;2.01)	0.95 (0.61;1.48)	1.19 (0.65;2.18)	0.81 (0.48;1.38)
all samples:	0.98 (0.63;1.52)	1.21 (0.86;1.72)	0.98 (0.59;1.64)	0.99 (0.66;1.48)

relative risk with 95% confidence interval				

*p53 *gene SNP72				
	event-free survival		overall survival	

	Arg/Arg vs. Arg/Pro and Pro/Pro	Pro/Pro vs. Arg/Pro and Arg/Arg	Arg/Arg vs. Arg/Pro and Pro/Pro	Pro/Pro vs. Arg/Pro and Arg/Arg

DLBC samples	1.06 (0.69;1.65)	1.31 (0.40;4.30)	1.13 (0.68;1.88)	1.60 (0.37;6.90)
all samples:	0.93 (0.66;1.31)	1.45 (0.63;3.30)	0.99 (0.66;1.47)	1.41 (0.51;3.87)

relative risk with 95% confidence interval				

### p53 gene SNP72 G/C

The allelic frequencies of the *p53 *gene SNP72 G/C polymorphism have been reported to vary widely between the ethnicities [20]. For European/North American Caucasians the frequency of the minor (C) allele coding for proline at position 72 was between 22 and 30% in the various studies, comparable with the frequency observed in our controls (26%) [21], and references therein). Among the patients diagnosed with NHL or DLBCL, the C allele was found with a frequency of 25%, respectively. Again, all genotype frequencies were as expected under the Hardy-Weinberg equilibrium.

Since it was conceivable that our female patients ≤ 51 years of age at the time of diagnosis, instead of having a reduced p53 response due to the SNP309 G/G genotype as was observed in the Ashkenazi Jewish cohort [11], have an apoptosis-impaired p53 associated with the SNP72 C/C genotype [22], we analyzed the SNP72 genotype frequencies by the same methods. No significant difference was detected between female NHL or DLBCL patients diagnosed by 51 years of age and those diagnosed later (figure 3B). Specifically, in the NHL group, two of 31 women diagnosed by 51 years and three of 105 women diagnosed later, exhibited the C/C genotype. For women diagnosed with DLBCL, these numbers were 2/20 and 2/70, respectively.

Finally, we examined whether SNP72 G/C has an influence on prognosis. Kaplan-Meier plots were calculated for EFS and OS, stratified according to genotypes (figure 4B). Cox proportional hazard analysis to adjust for IPI-factors (age >60; ex. involvement >1; ECOG 2–4; Stage III-IV; LDH > ONV) and bulky disease showed no difference between the genotype groups C/C and G/G. (Again, an independent influence of SNP72 G/C on EFS/OS could not be detected in this multivariate analysis). The results of the Cox regression analyses are summarized in Table 3.

## Discussion

The levels of the p53 tumor suppressor are primarily controlled by the E3 ubiquitin ligase MDM2 [1]. *MDM2 *promoter polymorphism SNP309 G gives rise to higher levels of MDM2 in response to stress or estrogen, and consequently, inhibits p53 more efficiently than SNP309 T [8,10]. Several studies have implicated estrogen signalling manipulation in cancer incidence and progression (reviewed in [23]), and in gender-specific differences in DLBCL incidence (for example, [24]). In accord with these findings, Bond and colleagues have recently reported that women homozygous for SNP309 G were diagnosed with DLBCL on average 13 years earlier than women homozygous for the T allele (G/G women: 55 years; T/T women, 68 years), whereas men did not show such a correlation. They also documented that the G/G genotype is significantly more frequent among women diagnosed before menopause (10/21 G/G-individuals were diagnosed by age 51 vs. 0/21 T/T-individuals; 11/58 G/G women were diagnosed by age >51 vs. 13/58 T/T women), and suggested that estrogen may co-operate with the G allele to accelerate lymphoma formation [11]. These findings could not be confirmed by our study; we observed only 3/20 G/G women diagnosed by age 51. In this context it should be noted that Bond et al. observed 24% G/G homozygotes among 976 healthy Caucasians of Ashkenazi Jewish descent, whereas we observed 14% in our healthy cohort of 512 central European Caucasians. Thus and in accord with these authors, Ashkenazi Jewish Caucasians have a significantly higher SNP309 G/G genotype frequency than Non-Ashkenazi Caucasians from central Europe (p < 0.001; Fisher's exact test).

*MDM2 *SNP309 T/G has originally been reported to be associated with an increased risk for tumor formation in patients with an inherited mutated p53 allele (Li-Fraumeni syndrome) and in patients with sporadic soft tissue sarcoma [10]. It should be kept in mind though that MDM2 is a pleiotropic E3 ubiquitin ligase with many cellular targets besides p53, one of the latest on the list being topoisomerase II [25]. SNP309 might therefore affect several cancer-relevant pathways. Homozygosity for SNP309 G has been linked to a significantly earlier onset of several hereditary and sporadic cancers, including breast carcinomas and osteosarcomas, but also to DLBCL, adult soft tissue sarcoma, invasive ductal breast cancer, and colorectal cancer specifically in women. The polymorphism has furthermore been associated with uterine leiomyosarcoma, squamous cell carcinoma of the head and neck, the outcome of breast cancer, non-small cell lung cancer, hepatocellular carcinoma in patients with chronic hepatitis C, gastric carcinoma, esophageal squamous cell carcinoma, nasopharyngeal carcinoma, ovarial carcinoma, sporadic endometrial cancer, invasive bladder cancer, and renal cell carcinoma (for a recent review, see [8]). However, other studies fail to show an association. For instance, age of onset of colorectal cancer in Lynch syndrome, lung cancer risk in a Chinese population, lung cancer risk, incidence of breast cancer in mutant BRCA1 carriers, incidence of breast and ovarian cancer, breast cancer risk in a Chinese population, breast cancer risk, age at diagnosis of HNPCC patients, basal cell carcinoma risk, risk and prognosis of glioblastoma (reviewed in [8]), and finally NHL and DLBCL in Non-Ashkenazi European Caucasians (this study), were not associated with SNP309. This together with the discrepancy between our findings and that of Bond and colleagues on DLBCL [11], points to the importance of other genetic modifiers in the p53 pathway.

Since its first description 20 years ago, hundreds of studies on the *p53 *gene SNP72 polymorphism and cancer susceptibility have been completed. Positive correlations were reported, for instance, for hepatocellular carcinoma [26], non-polyposis hereditary colorectal cancer [27,28], nasopharyngeal carcinoma [29], and melanoma [30]. Recent meta-analyses found positive correlations of the C/C genotype with gastric cancer in Asians [31], esophageal cancer [32], and overall cancer mortality [33]. By contrast, other meta-analyses failed to find a correlation with lung cancer [34] and breast cancer [20]. No correlation was also found for acute myelogenous leukaemia [35] and for multiple myeloma, except when studied in combination with other polymorphisms [36]. Likewise, our study on patients with NHL and DLBCL failed to establish a correlation with SNP72 G/C. Neither *MDM2 *gene SNP309 T/G nor *p53 *gene SNP72 G/C influences diffuse-large B-cell lymphoma in central European Caucasians.

## Conclusion

We found no evidence that *MDM2 *gene SNP309 or *p53 *gene SNP72 is associated with an increased risk for, or accelerated formation of, diffuse-large B-cell lymphoma in men or women of central European Caucasian ethnicity. Furthermore, neither SNP309 nor SNP72 was correlated with age of onset, diagnosis, or survival of patients. These polymorphisms may thus act as genetic modifiers in dependence of a population genetic background.

## Competing interests

The authors declare that they have no competing interests.

## Authors' contributions

JB, FP, AW, MM and LK carried out the probe sampling and genetic analyses, MK performed the statistical analysis and helped with the manuscript, LT and MP collected and characterized the patients and participated in the design of the study and manuscript preparation, OL and AM designed and optimized the genotyping, KR conceived of the study, supervised the laboratory work, and prepared the manuscript. All authors read and approved the final manuscript.

## Pre-publication history

The pre-publication history for this paper can be accessed here:



## References

[B1] Bond GL, Hu W, Levine AJ (2005). MDM2 is a central node in the p53 pathway: 12 years and counting. Curr Cancer Drug Targets.

[B2] Mendrysa SM, O'Leary KA, McElwee MK, Michalowski J, Eisenman RN, Powell DA, Perry ME (2006). Tumor suppression and normal aging in mice with constitutively high p53 activity. Genes Dev.

[B3] Jones SN, Roe AE, Donehower LA, Bradley A (1995). Rescue of embryonic lethality in Mdm2-deficient mice by absence of p53. Nature.

[B4] Montes de Oca Luna R, Wagner DS, Lozano G (1995). Rescue of early embryonic lethality in mdm2-deficient mice by deletion of p53. Nature.

[B5] Oliner JD, Kinzler KW, Meltzer PS, George DL, Vogelstein B (1992). Amplification of a gene encoding a p53-associated protein in human sarcomas. Nature.

[B6] Leach FS, Tokino T, Meltzer P, Burrell M, Oliner JD, Smith S, Hill DE, Sidransky D, Kinzler KW, Vogelstein B (1993). p53 Mutation and MDM2 amplification in human soft tissue sarcomas. Cancer Res.

[B7] Freedman DA, Levine AJ (1999). Regulation of the p53 protein by the MDM2 oncoprotein--thirty-eighth G.H.A. Clowes Memorial Award Lecture. Cancer Res.

[B8] Bond GL, Levine AJ (2007). A single nucleotide polymorphism in the p53 pathway interacts with gender, environmental stresses and tumor genetics to influence cancer in humans. Oncogene.

[B9] Kinyamu HK, Archer TK (2003). Estrogen receptor-dependent proteasomal degradation of the glucocorticoid receptor is coupled to an increase in mdm2 protein expression. Mol Cell Biol.

[B10] Bond GL, Hu W, Bond EE, Robins H, Lutzker SG, Arva NC, Bargonetti J, Bartel F, Taubert H, Wuerl P, Onel K, Yip L, Hwang SJ, Strong LC, Lozano G, Levine AJ (2004). A single nucleotide polymorphism in the MDM2 promoter attenuates the p53 tumor suppressor pathway and accelerates tumor formation in humans. Cell.

[B11] Bond GL, Hirshfield KM, Kirchhoff T, Alexe G, Bond EE, Robins H, Bartel F, Taubert H, Wuerl P, Hait W, Toppmeyer D, Offit K, Levine AJ (2006). MDM2 SNP309 accelerates tumor formation in a gender-specific and hormone-dependent manner. Cancer Res.

[B12] Bond GL, Menin C, Bertorelle R, Alhopuro P, Aaltonen LA, Levine AJ (2006). MDM2 SNP309 accelerates colorectal tumour formation in women. J Med Genet.

[B13] p53-knowledgebase http://p53.bii.a-star.edu.sg/aboutp53/snps/snpdetail.php?geneid=X54156&snppos=12139.

[B14] Hong Y, Miao X, Zhang X, Ding F, Luo A, Guo Y, Tan W, Liu Z, Lin D (2005). The role of P53 and MDM2 polymorphisms in the risk of esophageal squamous cell carcinoma. Cancer Res.

[B15] Pfreundschuh M, Trumper L, Kloess M, Schmits R, Feller AC, Rube C, Rudolph C, Reiser M, Hossfeld DK, Eimermacher H, Hasenclever D, Schmitz N, Loeffler M (2004). Two-weekly or 3-weekly CHOP chemotherapy with or without etoposide for the treatment of elderly patients with aggressive lymphomas: results of the NHL-B2 trial of the DSHNHL. Blood.

[B16] Pfreundschuh M, Trumper L, Kloess M, Schmits R, Feller AC, Rudolph C, Reiser M, Hossfeld DK, Metzner B, Hasenclever D, Schmitz N, Glass B, Rube C, Loeffler M (2004). Two-weekly or 3-weekly CHOP chemotherapy with or without etoposide for the treatment of young patients with good-prognosis (normal LDH) aggressive lymphomas: results of the NHL-B1 trial of the DSHNHL. Blood.

[B17] Boltze C, Roessner A, Landt O, Szibor R, Peters B, Schneider-Stock R (2002). Homozygous proline at codon 72 of p53 as a potential risk factor favoring the development of undifferentiated thyroid carcinoma. Int J Oncol.

[B18] Pont-Kingdon G, Lyon E (2005). Direct molecular haplotyping by melting curve analysis of hybridization probes: beta 2-adrenergic receptor haplotypes as an example. Nucleic Acids Res.

[B19] Ihaka R, Gentleman RR (1996). A language for data analysis and graphics. J Comp Graph Statistics.

[B20] Weston A, Godbold JH (1997). Polymorphisms of H-ras-1 and p53 in breast cancer and lung cancer: a meta-analysis. Environ Health Perspect.

[B21] Pietsch EC, Humbey O, Murphy ME (2006). Polymorphisms in the p53 pathway. Oncogene.

[B22] Dumont P, Leu JI, Della Pietra AC, George DL, Murphy M (2003). The codon 72 polymorphic variants of p53 have markedly different apoptotic potential. Nat Genet.

[B23] Dietel M, Lewis MA, Shapiro S (2005). Hormone replacement therapy: pathobiological aspects of hormone-sensitive cancers in women relevant to epidemiological studies on HRT: a mini-review. Hum Reprod.

[B24] Nelson RA, Levine AM, Bernstein L (2001). Reproductive factors and risk of intermediate- or high-grade B-Cell non-Hodgkin's lymphoma in women. J Clin Oncol.

[B25] Nayak MS, Yang JM, Hait WN (2007). Effect of a single nucleotide polymorphism in the murine double minute 2 promoter (SNP309) on the sensitivity to topoisomerase II-targeting drugs. Cancer Res.

[B26] Yu MW, Yang SY, Chiu YH, Chiang YC, Liaw YF, Chen CJ (1999). A p53 genetic polymorphism as a modulator of hepatocellular carcinoma risk in relation to chronic liver disease, familial tendency, and cigarette smoking in hepatitis B carriers. Hepatology.

[B27] Jones JS, Chi X, Gu X, Lynch PM, Amos CI, Frazier ML (2004). p53 polymorphism and age of onset of hereditary nonpolyposis colorectal cancer in a Caucasian population. Clin Cancer Res.

[B28] Kruger S, Bier A, Engel C, Mangold E, Pagenstecher C, von Knebel Doeberitz M, Holinski-Feder E, Moeslein G, Schulmann K, Plaschke J, Ruschoff J, Schackert HK (2005). The p53 codon 72 variation is associated with the age of onset of hereditary non-polyposis colorectal cancer (HNPCC). J Med Genet.

[B29] Tsai MH, Lin CD, Hsieh YY, Chang FC, Tsai FJ, Chen WC, Tsai CH (2002). Prognostic significance of the proline form of p53 codon 72 polymorphism in nasopharyngeal carcinoma. Laryngoscope.

[B30] Stefanaki I, Stratigos AJ, Dimisianos G, Nikolaou V, Papadopoulos O, Polydorou D, Gogas H, Tsoutsos D, Panagiotou P, Kanavakis E, Antoniou C, Katsambas AD (2007). p53 codon 72 Pro homozygosity increases the risk of cutaneous melanoma in individuals with dark skin complexion and among noncarriers of melanocortin 1 receptor red hair variants. Br J Dermatol.

[B31] Zhou Y, Li N, Zhuang W, Liu GJ, Wu TX, Yao X, Du L, Wei ML, Wu XT (2007). P53 codon 72 polymorphism and gastric cancer: A meta-analysis of the literature. Int J Cancer.

[B32] Hiyama T, Yoshihara M, Tanaka S, Chayama K (2007). Genetic polymorphisms and esophageal cancer risk. Int J Cancer.

[B33] van Heemst D, Mooijaart SP, Beekman M, Schreuder J, de Craen AJ, Brandt BW, Slagboom PE, Westendorp RG (2005). Variation in the human TP53 gene affects old age survival and cancer mortality. Exp Gerontol.

[B34] Matakidou A, Eisen T, Houlston RS (2003). TP53 polymorphisms and lung cancer risk: a systematic review and meta-analysis. Mutagenesis.

[B35] Zhang W, Hu G, Deisseroth A (1992). Polymorphism at codon 72 of the p53 gene in human acute myelogenous leukemia. Gene.

[B36] Ortega MM, Honma HN, Zambon L, Lorand-Metze I, Costa FF, De Souza CA, Lima CS (2007). GSTM1 and codon 72 P53 polymorphism in multiple myeloma. Ann Hematol.

